# Point prevalence survey of healthcare-associated infections in Slovakia: from zero to real data

**DOI:** 10.1186/2047-2994-4-S1-P277

**Published:** 2015-06-16

**Authors:** M Stefkovicova, I Rovny, J Brnova

**Affiliations:** 1 School of Health Care, Alexander Dubcek University of Trencin, Trencin, Slovakia; 2 School of Public Health, Slovak Medical University, Bratislava, Slovakia; 3 School of Health Care, The Catholic University Ruzomberok, Ruzomberok, Slovakia; 4 Laboratory of Molecular Microbiology, St. Elisabeth University, Bratislava, Slovakia; 5 Department of Laboratory Medicine, School of Health Sciences and Social Work, Trnava University, Trnava, Slovakia

## Introduction

Inadequate compliance to surveillance systems and lack of infection control professionals still exist in Slovakia, making it difficult to accurately assess the present burden of health care-associated infections (HAI).

## Objectives

In this study we presented data from European Centre for Disease Prevention and Control (ECDC) point prevalence study of HAI in Slovakia in comparison with data from Slovak mandatory incidence surveillance systems.

## Methods

Point prevalence survey of HAI was carried out according to a standardized methodology developed by the ECDC at 40 hospitals in Slovakia providing acute health care in June 2012. Data were collected at the country level, hospital level and the patient level according to standard protocol.

## Results

From 8397 patients included in the survey, HAI occurred in 298 (3,5 %; 2,7 % - 4,6 %) patients. The highest prevalence of HAI was found on the intensive care units (12,4 %). The most common types of HAI were urinary tract infections (26,2%), pneumonia and other lower respiratory tract infections (22,0 %), surgical site infections (15,7 %), bloodstream infections (9,9 %), infections of the eye, ear, upper respiratory tract infections (8,3 %) and skin and soft tissue infections (5,2%). The most frequent isolated microorganisms were *Escherichia coli* (15,0 %), *Klebsiella spp*. (12,5 %) and *Pseudomonas aeruginosa* (10,8 %). More than half of observed patients (60,5%) had at the time of monitoring introduced invasive medical device: central vascular catheter (3,4 %), peripheral vascular catheter (40,8 %), urinary catheter (14,1 %) or were intubated (2,1 %). According to data from Slovak mandatory incidence surveillance systems, HAI occurred long time only in 0,5 % of hospitalised patients.

## Conclusion

Improving compliance to surveillance systems of health care-associated infections represents an important and growing challenge in Slovakia. In this setting we urgently need to establish infection control teams in Slovak health care facilities according ECDC standards.

## Disclosure of interest

None declared.

